# “From Charlemagne to the Title of the King”: Political novel between estrangement and recognition

**DOI:** 10.12688/openreseurope.19920.1

**Published:** 2025-06-06

**Authors:** Zvonimir Glavaš

**Affiliations:** 1 University of Zagreb Faculty of Humanities and Social Sciences, Zagreb, City of Zagreb, Croatia

**Keywords:** estrangement, recognition, politics of literature, political novel, polyphonic novel, dialogism

## Abstract

Starting from a critical reflection on certain elements of Rita Felski’s polemic with the “hermeneutics of suspicion”, the article focuses on what may appear to be a binary opposition between the concepts of estrangement and recognition. It aims to show that what appears from another perspective as the mutual exclusivity of these two concepts and their associated theoretical traditions gives room for their co-implication in a dialectical relationship and that such a relationship has an intrinsically political dimension. Subsequently, it is argued that the described dialectic of estrangement and recognition can provide a suitable basis for revisiting discussions on the politics of literature – inextricably linked to these concepts from the beginning – and consequently even help in the search for a viable concept of the political novel.

1.

According to Felski, recognizing oneself in a book is the fundamental step in both the epistemic and the ethical-political effect of a literary text on the reader. But because of the prevailing tendencies in the modern humanities, she argues, this ubiquitous experience is largely frowned upon. Even more than that, “engagement with recognition is hedged round with prohibitions and taboos, often spurned as unseemly, even shameful, seen as the equivalent of a suicidal plunge into unprofessional naïveté” ( 1 on page 26). The main culprit for this sorry state is the decades-long dominance of the hermeneutics of suspicion ( 2 on page 1, 20), or what she earlier ^1^ referred to as anti-Hegelian thought and epitomized by Emmanuel Levinas, Jacques Lacan and Louis Althusser.

Although the mentioned three undoubtedly deserve their share of the “blame”, the paradigm Felski criticizes is certainly much older. Her later book *The Limits of Critique* (2015) extends this genealogical framework, but the point remains unchanged: Approaches focused on estrangement are still “looking disapprovingly at the countless readers who persist in using these texts in unseemly or inappropriate fashion – identifying with characters, becoming absorbed in narratives, being struck by moments of recognition” ( 2 on page 29).

Felski is certainly not alone in her aversion to this alleged academic fashion; she herself refers to several authors whom she regards as her direct predecessors (see 2 on page 22). However, her dismissal of the estrangement-centered paradigm by the emphasis on identification can be countered by the opposite stance of many scholars throughout the decades – from Yury Tynyanov in 1924 ^3^ to Anna Kornbluh in 2017 ^4^ – who argue that literary theory has never actually overcome the dominance of various identification-based positivist currents.

It is not the intention of this article to take sides in a struggle between the two paradigms. Rather, it aims to show that the concepts of estrangement and recognition are not mutually exclusive, but rather necessarily co-implicated in a dialectical relationship. Moreover, since they are unavoidable points of reference in discussions of the politics of literature and the political novel, I will attempt to show that the elaboration of this co-implication can provide a suitable ground for revisiting certain earlier discussions of that matter and help in the search for a viable concept of the political novel.

2.

Although it is over a century old, very short and written by a 24-year-old, Viktor Shklovsky’s *Art as a Device* (1917) is still an unsurpassable reference when it comes to discussing this topic. According to Shklovsky ( 5 on page 6), the estrangement implies the de-automatization of perception through a burdened form and a protracted and laborious process of perception. Such a process of perception, although characterized as self-sufficient, does not end in an impasse: It leads to a changed understanding, to seeing something familiar in a new light. This is perfectly complementary to how Felski describes recognition, when claiming:

When we recognize something, we literally “know it again”; we make sense of what is unfamiliar by fitting it into an existing scheme, linking it to what we already know. Yet, as Gadamer points out, “the joy of recognition is rather the joy of knowing more than is already familiar”. ( 1 on page 25)

In other words, both Shklovsky’s estrangement and Felski’s recognition can be described as a phenomenon that is “simultaneously reassuring and unnerving”, which “brings together likeness and difference in one fell swoop” ( 1 on page 25).

From the singular event to the broader literary context, this peculiar interplay remains the same. As Shklovsky puts it, “a life of poetic work moves from seeing to recognition, from poetry to prose, from the concrete to the general, […] from Charlemagne to the title of king ( 5 on page 6)”. ^1^ This formulation later gave rise to the formalist theory of literary evolution, most fully formulated by Tynyanov ^6^, which has shown that estrangement is a product of complex synchronic and diachronic relations; it implies the literary tradition as its constitutive background and eventually becomes the literary tradition. Or as Jan Mukařovský put it a decade later: “A work of art is always an imperfect application of aesthetic norms” ( 7 on page 33); the process in which “the norm is constantly being violated” ( 7 on page 33) is the same process in which a new one is formed.

One is confronted with the typical dynamic of transgression, an influential, ubiquitous concept that Felski considers notorious in its contemporary form. But in the continuity from George Bataille’s works in the mid-war era to contemporary authors who use this concept as a crucial tool in their study of literature, the constitutive dependence of transgression on the boundaries it crosses and exposes has been emphasized again and again. In words from Foucault’s preface to Bataille’s *Transgression*:

The limit and transgression depend on each other […]: a limit could not exist if it were absolutely uncrossable and, reciprocally, transgression would be pointless if it merely crossed a limit composed of illusions and shadows. ( 8 on page 73)

Felski, on the other hand, is so averse to this dynamic that she hastens to warn against a paradox that is in fact not so paradoxical, and argues against the strawmen she eagerly sets up. She claims that anti-Hegelians become “embroiled in a version of the Cretan liar paradox”, for how could one know “that an act of misrecognition had taken place” if one is “barred from self-understanding” ( 1 on page 28). But what is overlooked is that we are facing different levels (individual vs. collective practices) and different approaches (immediate vs. mediated); that the blind spots of one recognition are revealed by the parallax of the other. One could hardly seriously argue that Lacan or Althusser have completely negated the processes of (self-)recognition; the truth is rather that they have questioned their Cartesian variants.

Even more curiously, Felski argues that anti-Hegelian thought neglects “the dialogic and non-identitarian dimensions of recognition, as anchored in intersubjective relations that precede subjectivity” ( 1 on page 30), but there is no obstacle to apply this rather opaque characterization to theories of subjectivity within the “hermeneutics of suspicion”, nor does this necessarily preclude suspicion towards the appropriative components of recognition. Finally, and most importantly, even the literary examples Felski uses to emphasize the importance of recognition clearly imply estrangement in equal measure.

For example, when she discusses the political impact of Hendrik Ibsen’s *Hedda Gabler* (1890) on female readership, Felski explicitly states that “the experience of self-recognition and heightened self-awareness is routed through an aesthetic medium; to see oneself as Hedda Gabler is in some sense to see oneself anew” ( 1 on page 35). In a further elaboration, she introduces the distinction between two types of recognition: self-intensification and self-extension ( 1 on page 39), i. e. the recognition of immediate similarities and the construction of analogies between phenomena that appear disparate at first sight. Felski considers the latter to be a far more complex case and illustrates it with the example of Pankaj Mishra’s *The Romantics* (1999). In it, the echoes of Gustave Flaubert’s *Sentimental Education* (1869) draw unforeseen analogies between contemporary Benares and nineteenth-century France. The situation goes far beyond mechanical pairing of semblances; literature does not merely passively reflect a certain reality but intervenes to enable us to see it anew. This is analogous to the excerpts from Tolstoy offered as examples in Shklovsky’s *Art as a Device* (see 5 on page 7–8). To pick out only the most famous: The perspective of the horse in *Kholstomer* (1886), which exposes the irrationality of certain social conventions by allowing us to perceive human reality through the eyes of a non-human animal, or the chaotic description of battles in *War and Peace* (1869) which undermine the Clausewitzian perspective on war.

Finally, something else is striking about this analogy: Not only are the examples offered by Felski and Shklovsky structurally similar, but the more similar they become, the more politically potent they are. The intrinsically political dimension of this estranged recognitions was recognized by both to a certain extent, ^2^ but neither approached it more structurally by raising the question of the politics of literature.

3.

The issue of the politics of literature has long been viewed primarily through the prism of engaged literature and conceptualized with an emphasis on recognition rather than estrangement. The laudable exception is the range of theoretical approaches that affirm a form of ideological critique in conjunction with reading against the grain to show how literature exposes various ideological mechanisms, most of which Felski has in mind when she speaks of “hermeneutics of suspicion”. Genealogically speaking, such a grouping is not wrong, even if certain nuances need to be distinguished. One of these concerns the degree of agency granted to literature. This question is particularly interesting when considering the examples given by Shklovsky and Felski, as they imply the ability of literature not only to reflect social contradictions, but also to substantially change the reader’s perspective.

One of the more recent approaches kin to this position is that of Rancière. According to him, the politics of literature is “not the politics of writers” nor the mere “representation of social structures, political movements or various identities in literary works” ( 10 on page 11). Rather, by “politics of literature” we should understand a literature that engages with politics “by remaining literature”; that is partaking in the division of the sensible, making visible what was invisible, giving a voice to those who had none etc. ( 10 on page 12).

This perspective is derived from Rancière’s general definition of politics, most famously expressed in *Disagreement* (1999). In contrast to conventional terms, he uses the term “police” to designate the procedures that organize power and distribute places and roles in a given system ( 11 on page 28), while “politics” is reserved for whatever activity that “shifts a body from the place assigned to it or changes a place’s destination. It makes visible what had no business being seen, and makes heard a discourse where once there was only place for noise” ( 11 on page 30).

Although interventions in the order of the sensible could take different guises, it is clear from the examples in Rancière’s analyses that he focuses mainly on the formal rather than the thematic level. This is particularly true of his later works, in which he largely confines himself to subversions of the principle of appropriate representation by the equality of themes and styles – the famous democracy of writing ( 10 on page 21). This tendency has been sharply criticized by several critics who objected to his preference for modernist devices and allegedly inflationary backlash of his overly heroic vision of literature. According to them, if everything can be seen as political, then nothing really is. Moreover, ascribing an intrinsic politicalness to (modernist) literature leads to a paradoxical asymmetry in which what should be emancipatory becomes privileged, while at the same time emancipatory agency of conventional politics loses its significance.

Even though some of these critiques can be accused of a certain reductionism themselves – occasionally neglecting the differences between literariness, literature as institution, various artistic regimes (see 12 and 13) and the opposition between the disagreement ( *la mésentente*) and the misunderstanding ( *le malentendu*) – the question of whether Rancière’s overemphasis on formal estrangement has rather blunted or sharpened the blade of his criticism is a reasonable one. In some cases, Rancière is on the edge of falling into the trap previously fallen into by another theorist with a heroic vision of literature. Ironically, it was Rancière ( 14 on page 153, 163) who criticized Gilles Deleuze’s inconsistent affirmation of – in Deleuzian terms, later accepted by Rancière – the molecular subversion of the mimetic, but above all the impossibility of linking Deleuzian ontology with Deleuzian politics; the molecular dissolution of the paternal order with the establishment of another community, albeit a fraternal one. ^3^

This enthusiastic but problematic overemphasis on estrangement can be balanced by a little recognition, in a gesture similar to one from the previous chapter. In his *Mute Speech*, Rancière ( 25 on page 124, 134) has shown that the radical democracy of writing puts literature in danger of two possible extremes: either dissolution into ordinary speech or complete hermetic closure. And he is not the only one to issue a similar warning.

Although Derrida, for example, also defines literature as the institution that “allows one to say everything, in every way” ( 15 on page 36), which is crucial for its political dimension, he emphasizes that he does not equate the progressive effects of literature with absolute avant-gardism:

I do not believe it is enough […] to persist stubbornly to smuggle in super-refined avantgarde products without struggling for a massive transformation of the apparatuses. But neither is it useless. […] Thus, one must […] work in several directions, in several rhythms. The monorhythmic and the monocode always spell immediate reappropriation. […] One must tamper with the code, its homogeneity and the singularity of the system that orders and regulates languages and actions. ( 16 on page 58) ^4^

This seemingly paradoxical relationship between invention and convention ( 15 on page 316) can be taken – as Shklovsky anticipated ( 5 on page 6) – as analogous with the relationship between the singular and the universal. Derrida repeatedly pointed – most famously in *Before the Law* ( 15 on page 213) – to literature as a peculiar institution that reveals the paradoxical relationship between the universal and the singular; i. e. a contingent grounding of the supposedly always already grounded universal and the repetition of the supposedly unrepeatable singular. This is indeed a terrain on which literature encounters the law and exposes it.

The dual nature of this catachrestic connection is what might be missing in Rancière’s or Deleuze’s politics of literature. The overemphasis on misunderstanding should be corrected by disagreement. The unmasking and undoing of the catachrestic grounding is present, but not the other, subsequent catachrestic grounding. Although the center of structure is an illusion, the non-centered structure is unthinkable ( 11 on page 352), which is why it is difficult to think of molecular disorder as politics.

Rancière’s *Disagreement* (1999) takes this into account. Politics there is described as a rare moment in which a differential system is exposed as contingent and called into question. The eponymous disagreement, however, always takes place between actors who find themselves between subjectivation and identification ( 11 on page 37). The request of those who are the miscount of a communal count always has a subject, and that subject always aims to transcend its particular position. The crucial role of literariness in this process is not neglected. Rancière argues that “the modern political animal is first a literary animal, caught in the circuit of a literariness that undoes the relationships between the order of words and the order of bodies that determine the place of each” ( 11 on page 37). But in moving from the literariness of politics to the politics of literature, he slips from what Oliver Marchart calls the post-foundational closer to the anti-foundational paradigm. ^5^

Is there a way to avoid that step and to reconcile estrangement and recognition (subjectivation and identification) on this terrain? One might not need to reinvent the wheel to do so; a return to the early twentieth century might suffice. In his *Marxism and the Philosophy of Language*, Valentin Voloshinov anticipated certain poststructuralist insights by arguing that since “various different classes will use one and the same language”, “differently oriented accents intersect in every ideological sign” and thus, “sign becomes an arena of the class struggle” ( 21 on page 23). However, the poly-accentuated nature of signs normally only makes itself felt in times of social crisis ( 21 on page 24). But in literature, the accentuation is explicitly verbalized in a kind of an estranging effect. It is a “more ingrained kind of *evaluation via form* that finds expression in the very manner in which the artistic material is viewed and deployed” ( 21 on page 184). This “social multiaccentuality” is precisely the same phenomenon that lies at the heart of Bakhtin’s dialogic character of novelistic discourse when he writes that “for the writer of artistic prose, […] the object reveals first of all precisely the socially heteroglot multiplicity of its names, definitions and value judgments” ( 22 on page 278).

Novelistic heteroglossia, however, not only reveals social heterogeneity, thereby undermining the status of dominant discourse, but also enables identification with various positions. This already emerges in Bakhtin’s *Problems of Dostoevsky’s Poetics* (1984), in which the identification of readers with often contradictory, but equivalent points of view forms the starting point of the entire analysis.

Bakhtin and Voloshinov succeed in bringing together what in contemporary discussions often appear to be polemically opposed positions. These include approaches that favor the experience of individual and/or collective recognition based on a sort of reflection, approaches that focus on literature exposing false recognition and social contradictions in its background, and approaches that emphasize the emancipatory power of literature through various forms of estrangement. This synthetic capacity of the Bakhtin circle stems from the fact that it succeeded in achieving what, according to Jurij Striedter ( 9 on page 76), remained unfinished in the confrontation between formalism and Marxism – namely, the combination of formal and socio-ideological criticism.

It is therefore not surprising that a similar attempt can be observed in the works of Fredric Jameson, fueled by his conviction that Marxist criticism should accept validity of “rival” approaches at a local level, with Marxism as an insurmountable horizon ( 24 on page x). Jameson condenses entanglement of estrangement and recognition in his concepts of the political unconscious and history as an absent cause available only through narrativization ( 24 on page 20), along with the dialectic of ideology and utopia ( 24 on page 281). It links Voloshinov’s “arena of class struggle” to the emancipatory impulses, and historicises them both. But his persistent historicization is a double-edged sword: It allows him to contextualize the utopian and expose it as a catachrestic claim to universality, but his readings, in turn, remain largely limited to the original historical horizon of the artwork and place less emphasis on the later contexts of reception.

Finally, speaking of history, one of the better examples of a multifaceted engagement with the politics of literature comes not from a literary scholar, but from a historian. In the introduction to his *History, Politics, and the Novel* (1987), LaCapra announces his intention to “mediate and supplement the relation between formal and thematic interpretation” ( 25 on page 1), and derives three possible forms that the relationship between literature and history can take: symptomatic, critical, and transformative. The first is a more or less sophisticated version of the theory of reflection, in which the literature merely reproduces established ideological patterns. In the second two cases, on the other hand, the literary text is granted more agency, as it either reveals or even partially undermines latent ideological mechanisms. It is thus not surprising that one of the most important influences on LaCapra comes from Bakhtin’s theory of polyphony (see 25 on page 9).

Be that as it may, one last question remains: If there are theoretical approaches that recognize the specific dynamics of estrangement and recognition in the field of the politics of literature – what implications does this have for the question of genre? Is it possible to talk about the political novel, or are we limited to recognizing political aspects in individual literary works? Is it possible to go through the Scylla and Charybdis of generalization and individualization – not to outline an all-encompassing genre, but neither one that is too narrow?

4.

The problem of politics is fundamentally a problem of homonymy, and the politics of literature adds another level of homonymy to the basic one. Finally, the third arises with the term ‘political novel’ as a frequently used but rarely theorized buzzword, resulting in a proliferation of seemingly synonymous and self-explanatory terms that are in fact neither. It is one thing to say that a novel produces political effects, and another to assume that it shares structural features with other novels that might best be treated collectively under the label of the political novel.

The first possible approach to this issue is the purely thematic one. One can say that the political novel is a novel that explicitly narrates political structures, actors and struggles. But even if this were completely unambiguous, which it is not, the question remains as to how methodologically wise it is to define a genre strictly on the thematic level. ^6^ Despite some undeniable advantages of the thematic approach, the danger of it slipping into one-dimensional positivist approaches that ignore the literariness of the literary text is much greater. The need to go beyond the purely thematic approach is already evident in the introductions to the few studies of the political novel that exist to date. However, although they have formulated useful entry points, their subsequent analyses have mostly slipped back into thematic criticism (see 26).

Ironically, some of the best observations are found in Irving Howe’s *Politics and the Novel*, although it begins with the assertion that he does not believe in the political novel as a genre because in this case it is not possible to “mark any fundamental distinctions of literary form” ( 27 on page 18). Rather, he argues, this category can at best “point to a dominant emphasis, a significant stress in the writer’s subject or in his attitude toward it” ( 27 on page 18). The validity of Howe’s assertion, however, depends largely on how rigidly one conceives of genre. Since this “dominant emphasis” must be realized through some formal device, it can easily be translated into the formalist concept of the dominant. And it is the dominant that is crucial to the determination of genre.

Nevertheless, one of Howe’s main theses is that the political novel is a novel “in which political ideas play a dominant role or in which the political milieu is the dominant setting” ( 27 on page 19). More importantly, he argues that “like a nimble dialectician, the political novelist must be able to handle several ideas at once, to see them in their hostile yet interdependent relations and to grasp the way in which ideas *in the novel* are transformed into something other than the ideas of the political program” ( 27 on page 23). The ideas that the novel “appropriates are melted into its movement and fused with the emotions of its characters”, which “stir characters into passionate gestures and sacrifices” ( 27 on page 23). It may seem that this description remains encapsulated at the thematic level, but an attentive reader will recognize familiar topoi that suggest that Howe was directly or indirectly influenced by Bakhtin. This is all the more true as he singles out Fyodor Mikhailovich Dostoevsky’s *The Possessed* as “the greatest of all political novels” ( 27 on page 24).

In his commentary on Boris Engelhardt’s *Dostoevsky’s Ideological Novel* (1924), Bakhtin argues that Engelhardt was the first to correctly define the role of the idea in Dostoevsky’s novels. According to him, the idea here is neither a “principle of representation (as in any ordinary novel), nor the leitmotif of representation, nor a conclusion drawn from it (as in a novel of ideas, or a philosophical novel); it is, rather, the object of representation” ( 29 on page 24). These objects are at the same time the articulating principles of characters’ worldviews. Ideas, however, are never “ideas in themselves”, but are embodied in consciousnesses in interaction with other consciousnesses, in their historical context ( 29 on page 31).

Another point on which Howe comes close to Bakhtin is his attempt to distinguish the political novel from the social novel. While the latter “always presupposed a substantial amount of social stability” ( 27 on page 21), which enables it to vivisect the society under its glass, the former takes place when “the *idea of society*, as distinct from the mere unquestioned workings of society, has penetrated the consciousness of the characters in all of its profoundly problematic aspects, so that there is to be observed in their behaviour […] some coherent political loyalty or ideological identification” ( 27 on page 21). According to Howe, this shift is characteristic of times in which social stability gives way to the questioning of the foundations of the respective society and attention shifts from “the gradations within society to the fate of society itself” ( 27 on page 21).

This is exactly the same situation that, according to Bakhtin, causes the irreducible plurality in Dostoevsky’s novels. However, it is very important to keep in mind that “the essential dialogicality of Dostoevsky” is not limited to the characters: “ *The polyphonic novel is dialogic through and through*”, i. e. the contrapuntal relationships “exist among all elements of novelistic structure” ( 29 on page 40). Polyphonic novel such as Dostoevsky’s is, however, a very specific construction; it may not be wise to limit the concept of the political novel to solely this form. Rather, in line with Bakhtin’s later perspective ^22^, I propose to treat novelistic polyphony as a spectrum, and the polyphonic novel more narrowly as a particularly good example of the situation in which the dominant of a novel is to expose a particular social paradigm as contingent and to challenge it by focussing on the clash between it and what is denied a voice of its own. ^7^ In this way, it would simultaneously imply both estrangement and recognition.

But what is the precondition for such a combination of estrangement and recognition that allows certain elements to have a collective rather than an individual significance? Is there a discrete boundary between the social and the political novel? There is no definitive answer to this, and the question is inevitably dependent on context and reception. To assert that, however, is not tantamount to radical relativism. Rather, it is a retreat to a consistent historization.

As Mukařovský argues, the sociological observation shows us that “the work of art itself is not a constant”, and that every “shift in time, space or social surroundings alters the existing artistic tradition through whose prism the art work is observed” ( 7 on page 60). He therefore distinguishes between the artwork as a material artifact and the artwork as an aesthetic object. Consequently, Mukařovský argues that if the independent aesthetic value of an artwork is determinable at all, it must be related to the artwork as an artifact and the dynamic unity of the extra-aesthetic values it contains, which meet the extra-aesthetic values of the perceiver and lead to complex confrontations ( 7 on page 85–87). The more complex the artwork is as an artifact, the more likely it is to engage meaningfully (as an aesthetic object) with the readership over time and consequently to be perceived as a work with a higher and more enduring aesthetic value. Therefore, Mukařovský asserts that a contemporary reader of Dostoevsky is not interested in whether the story of Raskolnikov really happened. Rather he “feels the strong relationship of the novel to reality, and not only to that reality which is described in the novel, […] but to the reality which the reader himself is familiar with, […], to actions on the part of the reader which might have been caused by the situations” ( 7 on page 75). This remark, so reminiscent of Felski’s self-extension ( 1 on page 39), can serve as a valuable correction to the approaches that neglect the reception.

Similarly, it can be argued that the more complex a certain novel is as an artifact, the greater the chance that it will be recognized as a dramatization of a clash of different social paradigms and as a platform for subjectivation against one of them and identification with another. Certain novels have almost invariably been recognized as narrativisations of such disagreement, while others may lose or gain this status depending on the historical situation. But in both cases, this depends on the structural features of the novel as an artifact which are perceived as dominant in its aesthetic concretization. Of course, this does not mean that a novel recognized as political cannot belong to other genres at the same time.

This framework certainly has its blind spots, but it might strike a satisfactory balance between flexibility and explanatory power. This can be tested with a few examples. First, it allows us to understand why Dostoevsky’s *The Possessed* (1872) – as a novel dealing with the microcosm of a fictional town where the clash of political ideas stirs the revolt with destructive consequences – is invariably recognized as a political novel. At the same time, it can encourage the question whether all such readings of *The Possessed* take into account Bakhtin’s thesis about the presence of contrapuntal relations “among all elements of novelistic structure” ( 29 on page 24, 40), or whether they remain within the thematic level.

This difference becomes even more important in cases like Jonathan Littell’s *The Kindly Ones* (2006) as one of the best contemporary political novels. The confessions of a fictional SS officer, which lead the reader from one monstrous atrocity to another, deal with overtly political issues of criminal ideology and totalitarian state, and offer an unsettling mix of estrangement and recognition, largely due to the autodiegetic narration of a war criminal. However, the full political potential of the novel is not realized if components such as the mythological and historical subtext, the numerous literary allusions, the chapter headings and the tensions between different structural levels are disregarded.

Dystopian narratives such as George Orwell’s *Nineteen Eighty-Four* (1949) are another unavoidable example of the political novel with a similar dialectic of recognition and estrangement. Unlike the previous examples, its setting is not the present or the past, but the dystopian future. The estranging differences between this future and the actual present are at the same time bridges that lead to the recognition of analogies and hyperboles of certain contemporary phenomena. The obvious analogies and extrapolations lead to a pronounced ability to allegorise, which further emphasises the political relevance of the novel for the reader’s historical context. The situation is analogous with other similar allegorical narratives, such as Franz Kafka’s novels. The notable difference is that the allegorical potential of *The Trial* (1925) or *The Castle* (1926) largely transcends their thematic level. ^8^

In popular spy and crime novels such as the ones about James Bond (by Ian Flemming), George Smiley (by John Le Carré) or Gereon Rath (by Volker Kutscher) and their adaptations, things are less obvious. The reader could hypothetically focus only on the spy plot, but in most cases it is difficult to separate it from the political dynamics in its background – the dynamics of the disintegration of a stable order or the clash of the competing social paradigms in times of upheaval. Although their characters and narrative devices are simpler than those of Dostoevsky or Littell and more vivid than those of Orwell, they are less a means of narrating individual fates than political conflicts of their time.

This is less unambiguous in cases where the boundary between individual and collective recognition depends even more on the context of reception. However, the difference between the novel as artifact and as aesthetic object may help to explain Felski’s question ( 1 on page 44) of how a novel like Hall’s *Well of Loneliness* (1928) – which deals with issues of recognition conflicted with established social categories by telling a story of an early twentieth-century lesbian couple whose romance clashes with the social norms of the time – was at one time an important vehicle for collective identification and at another time became “the novel most hated by the lesbians themselves” ( 1 on page 44), perceived as stereotypical and restrictive. Similarly, contemporary examples such as Dino Pešut’s *Daddy Issues* (2020) – a Croatian gay generational *bildungsroman* that revolves around issues of gay identity in a conservative society and socio-economic injustices of the post-socialist state – and Olumide Popoola’s *When We Speak of Nothing* (2017) – a British coming-of-age novel that explores the intersection of generational, racial, (post)colonial and queer identity issues – both tell stories of individuals with which they provide a platform for estrangement and recognition that transcend the individual level in the current context, in which these parts without a part struggle for acknowledgment. However, since these novels are significantly less complex than *The Possessed* or *The Kindly Ones*, significantly less allegorical than *The Trial* or *Nineteen Eighty-Four*, and their plots are built more around individual lives than collective conflicts, the question is whether this collective dimension will continue to be as relevant and prominent in the future. The answer to this question depends on the tensions between the prevailing social paradigm and the values and identity components in these novels.

Moving from more prototypical examples to the margins of the category obviously highlights serious challenges and potential shortcomings, while diluting a stricter definition by resorting to contextual relativism. However, this can hardly be avoided in the matter at hand, at least if one simultaneously wants to avoid the pitfall of reductive positivism that attributes recognition to that in which no one recognizes himself, or estrangement to that which has long since ceased to be estranging. The dialectical relationship between estrangement and recognition is thus perhaps not a panacea, but its grasp seems quite wide in relation to the notorious elusiveness of the terms contained in the syntagma of the political novel – an elusiveness that is so strange and yet so recognisable.

## Ethics and consent

Ethical approval and consent were not required.

## Data Availability

No data associated with this article.

## Footnotes

^1^ Sher’s translation is not optimal in this part, as it reads: “from Charlemagne to Charles the Fat”. The original formulation continues the singular-general chain, since the Slavic word for king is derived from Charlemagne’s name.

^2^ Jurij Striedter argues that “ethical”, i. e. essentially political effect, was “unmistakable in the passages from Tolstoy discussed by Shklovsky” ( 9 on page 24), albeit secondary to the aesthetic one. It was also mainly perceived at the individual level, as a psychological effect. The latter is one of the main objections in Kornbluh’s critique of Felski: She praises Felski for emphasizing literary agency, but adds that “sourcing the crisis in method to our exhausted feelings about the mood of critique and proposing chipper feelings as the answer to crisis are both unfortunate psychologisms” ( 4 on page 398).

^3^ Deleuze introduced the opposition between the concepts of *molar* and *molecular* in his earlier works, most notably in *Anti-Oedipus* (1983) ^17^, and then applied them in his literary studies as well. Rancière’s critique of Deleuze primarily aimed at the most famous of them – *Kafka. Toward a Minor Literature* (1986) ^18^. However, despite articulating these critiques, Rancière adopts Deleuze’s *molecular* and *molar* as technical terms in his later analyses (see 12 and 10).

^4^ See Adorno’s complementary warning that art – while challenging “the lying positivism of meaning” – must preserve its communicativity ( 19 on page 191).

^5^ Marchart argues that post-foundational approach „does not attempt to erase completely such figures of the ground, but to weaken their ontological status”, which “does not lead to the assumption of the total absence of all grounds, but rather to the assumption of the impossibility of a *final* ground” ( 23 on page 2, 12). In contrast, the anti-foundational perspective is, according to him, a simple inversion of foundationalism, which assumes the non-existence of the ground whatsoever.

^6^ Wellek and Warren rejected such an approach, claiming that “a grouping based only on subject matter” might “go on endlessly” and encompass any number of possible themes ( 28 on page 242).

^7^ As Boyers argues, “A political novel […] will treat Being itself as problematic. Radical or conservative in outlook, it will project Being as an arrangement of objects, persons, and institutions in a world that has undergone change and may undergo change again. It is haunted by time because it concerns itself with arrangements that it knows to be impermanent, whatever the wishes of those who would like to believe otherwise” ( 30 on page 18).

^8^ Adorno argues: “Kafka, in whose work monopoly capitalism appears only distantly, codifies in the dregs of the administered world what becomes of people under the total social spell more faithfully and powerfully than do any novels about corrupt industrial trusts. The thesis that form is the locus of social content [ *Gehalt*] can be concretely shown in Kafka’s language” ( 31 on page 230).
