# Supplementary material for: “From Charlemagne to the Title of the King”: Political novel between estrangement and recognition
Source: Open Res Eur. 2025 Jun 6;5:155. [Version 1] doi: 10.12688/openreseurope.19920.1 (PMC12699207; doi:10.12688/openreseurope.19920.1)
Supplement: Peer review history

# Reviewer report: Reviewer response for version 1

**DOI:** 10.21956/openreseurope.21548.r64679

**Published:** 2025-12-11

**Authors:** Kristiawan Indriyanto

**Affiliations:** 1 Universitas Prima Indonesia, Medan, Indonesia

Overall this is a well organized paper, with only a minor adjustment needed.

 I think the abstract is a bit on the short side in which your whole argument is not well encapsulated. You may expand the abstract into around 100-150 words at least

 Please avoid using verbose words such as 'unsurpassable', be objective in your claim.

 Your main thesis/premise, the issue of the politics of literature which focuses on recognition takes far too long to be introduced (currently it is in the third section)

 The Bakhtinian concept of dialogism features in the keywords but it barely explained/alluded in your analysis

 The conclusion of your article is a bit abrupt and awkwardly placed, it seems too introduce a new concept instead of ending the discussion. Please rewrite it.

If any, are all the source data and materials underlying the results available?

Yes

Is the work original in terms of material and argument?

Yes

Is the work clearly and cogently presented?

Yes

Is the argument persuasive and supported by evidence?

Yes

Does the research article contribute to the cultural, historical, social understanding of the field?

Yes

Does it sufficiently engage with relevant methodologies and secondary literature on the topic?

Yes

Reviewer Expertise:

English literature, postcolonialism, environmental humanities

I confirm that I have read this submission and believe that I have an appropriate level of expertise to confirm that it is of an acceptable scientific standard.

# Reviewer report: Reviewer response for version 1

**DOI:** 10.21956/openreseurope.21548.r58429

**Published:** 2025-09-15

**Authors:** André P. DeBattista

**Affiliations:** 1 University of Malta, Msida, Malta

This study aims to analyze the "binary opposition between the concepts of estrangement and recognition", without taking sides in the "struggle between the two paradigms." Rather, the author aims "to show that the concepts of estrangement and recognition are not mutually exclusive, but rather necessarily co-implicated in a dialectical relationship." Overall, this paper manages to raise some very important points in a cogent manner.

 For example, the author makes a cogent point that the politics of literature is primarily viewed through the prism of "engaged literature and conceptualized with an emphasis on recognition rather than engagement". Citing Ranciere, the author also argues that the politics of literature is not the politics of the writers nor does it represents social structures and political movements, rather, the "politics of literature" is literature that engages with politics "by remaining literature." Commenting on the political novel, the author observes that "It is one thing to say that a novel produces political effects, and another to assume that it shares structural features with other novels that might best be treated collectively under the label of the political novel." In contrast to this, he cites Howe's argument that political novels necessarily have political ideas or the political milieu as a dominant role or setting. 

 This is an undoubtedly rich text which engages with several theoretical points and which is an interesting contribution to the field. My one suggestion, if I may, is to have properly labelled subheadings which would enhance the flow of the text.

If any, are all the source data and materials underlying the results available?

Yes

Is the work original in terms of material and argument?

Yes

Is the work clearly and cogently presented?

Yes

Is the argument persuasive and supported by evidence?

Yes

Does the research article contribute to the cultural, historical, social understanding of the field?

Yes

Does it sufficiently engage with relevant methodologies and secondary literature on the topic?

Yes

Reviewer Expertise:

My engagement is primarily on the link between politics and literature so, in terms of some of the theoretical points addressed, others may be far better at assessing their validity than myself.

I confirm that I have read this submission and believe that I have an appropriate level of expertise to confirm that it is of an acceptable scientific standard.
